# The consistency of antibiotic resistance’ results in two methods of disk diffusion and MIC in isolated organisms from patients with urinary tract infections

**Published:** 2015

**Authors:** A Karami, S Mazloom Zadeh, A Rastin, A Karami, P Shiri

**Affiliations:** * Infectious Disease Department, Zanjan University of Medical Sciences, Zanjan, Iran; ** Epidemiology Department, Zanjan University of Medical Sciences, Zanjan, Iran; *** Shahid Beheshti Hospital; Zanjan University of Medical Sciences, Zanjan, Iran; **** Cardiology Ward, Emam Ali Hospital; Kermanshah University of Medical Science, Kermanshah, Iran; ***** Zanjan University of Medical Sciences, Zanjan, Iran

**Keywords:** minimum inhibitory concentration, disk diffusion, urinary tract infection, drug resistance

## Abstract

**Introduction:** The urinary tract infection is the most common infection and drug resistance to it is increasing. Due to the acute infection, the prescribing of medicine is conducted before culture and antibiogram and according to the results, disk diffusion is adjusted. The aim of this study was to compare it with MIC to determine to what extent the current method could be useful.

**Methods:** This descriptive cross-sectional investigation research regarding drug resistance was conducted with the help of two methods of disk diffusion and MIC on the isolations of patients’ urine culture with UTI (midstream clean catch). Bacterial resistance was measured, and sensitivity and specificity were evaluated.

**Results:** The MIC method was considered the gold standard and, according to the related formula, the sensitivity and specificity of disk diffusion were related to 13 antibiotics obtained as it follows: ciprofloxacin 69% and 69.1% (0.0001 > p and Kappa = .292), cotrimoxazole 50% and 77.3% (p = 0.010), nitrofurantoin 84.7% and 58.2% (0.0001 > p and Kappa = 0.44), ampicilin 83.3% and 85.3% (0.0001 > p and Kappa = 0.33), ofloxacin 65.5% and 83.9% (0.0001 > p and Kappa = 0.429), cephalexin 46.2% and 75.2% (p = 0.012 and Kappa = 0.116), gentamicin 64.2% and 66% (0.0001 > p and Kappa = 0.276), ceftriaxone 27.6% and 80.9% (p = 0.216 and Kappa = 0.074), nalidixic acid 42.1% and 89.2% (0.0001 > p and Kappa = 0.354), imipenem 63.4% and 70.4% (0.0001 > p and Kappa 0.306), co-amoxiclav 83% and 71% (0.0001 > p and Kappa = 0.412), cefixime 21% and 80.9% (0.0001 > p and Kappa = 0.412), vancomycin 55.9% and 94.7 (0.9001 > p and Kappa = 0.532). Sensitivity and specificity of this method were reported to be lower than MIC.

**Conclusions:** Due to the low sensitivity and specificity of the disk diffusion method, antibiotic therapy should be certainly considered in clinical conditions, and risk factors for the infection and only by this approach cannot prescribe the drug.

## Introduction

Urinary tract infection is the most common primary disease, and annually, many people suffer from it in the world. Community-acquired urinary tract infection is the cause of referring to the clinics in America for nearly 7 million people [**1**]. Moreover, it is an important cause of mortality, especially in patients with immune deficiency [**2**].

Many microorganisms can infect the urinary tract, but the most common are the gram-negative bacilli. E. coli is the cause for about 80% of the acute infections in patients without catheters, urologic anomalies, and stone. Other gram-negative bacilli, particularly Klebsiella, Proteus, Enterobacter, Serratia, and Pseudomonas have a greater significance in recurrent infections and infections associated with urologic abnormalities, stone, or blockage and hospital infections and catheter-related [**1**].

Gram-positive cocci have fewer roles in the urinary tract infections. Staphylococcus saprophyticus is responsible for 15-10% of the acute infections symptoms in young women. Enterococci and Staphylococcus Aureus commonly cause diseases in patients with kidney stone history of surgery or instrumentation in the past [**1**]. The results of recent studies indicated that the bacteria resistance that causes the urinary tract infection first-line antibiotic is on the rise and one of the reasons for this increase is the improper and incorrect use of different antibiotics [**3**].

Drug resistance is different and often E. coli examples in America are resistant to amoxicillin, cephalexin, and sulfonamide and the resistance rate to co-trimoxazole is increasing. A high level of resistance to fluoroquinolones was reported in some countries [**4**]. Therefore, given the seriousness of the symptoms of urinary tract infection, prescribing drugs is done before attending urine culture and antibiogram, determining the strain that causes the disease. Particularly, their antibiotic resistance pattern in each area is necessary, being based on the most appropriate antibiotic options specified for treatment and so by prescribing a better drug preventing the creation of more antibiotic resistance.

One of the problems of the therapeutic infections is to build drug resistance. Drug resistance patients with previous antibiotic use, underlying diseases and immune weakness and community-acquired infections are more than ordinary people. There are different test ways to evaluate the sensitivity of organisms to antibiotics. The first method is to use the antibiotic disks (Kirby Bauer disk) and to evaluate sensitivity (S) or resistance (R) assessed by the measurement of the body growth area around the disc antibiotics [**5**].

The other method is the use of broth or agar dilution along with the addition of antibiotics and inoculated organisms. The lowest concentration of antibiotics, which causes the growth of the organism is determined (MIC = minimal inhibitory concentration) [**5**].

The third method is based on the E-test, that in this approach, MIC evaluated through bar containing antibiotic and agar medium (plate) that is impregnated with a certain amount of organisms [**5**].

In some hospitals, about 50% of Staphylococcus aureus and 90-80% of coagulase-negative Staphylococci are resistant to methicillin. Despite the sensitivity of the laboratory (in vitro) to drugs like cephalosporins, they may not be useful in the treatment of methicillin-resistant Staphylococcus, vancomycin being the free treatment. Clinical effectiveness depends on factors such as the drug concentration at the site of infection, the period of anti-bactericidal activity of antibiotics (Kinetic) and laboratory sensitivity of organisms. For example, Enterobacteriaceae resistant to tetracycline may respond to the treatment with tetracycline because the urinary concentration of the antibiotic is higher than the serum concentration of the drug [**5**].

## Materials and methods

The basis of the study was to investigate all the organisms from the urine culture of patients who were hospitalized in Valiasr Hospital with a diagnosis of urinary tract infection. UTI diagnosis was based on the symptoms of fever, chills, pain and suprapubic or flank area, burning and frequent urination, or symptoms of systemic and digestive infectious disease confirmed by the specialist. Urine culture was taken by the method of midstream clean catch and culture-positive was considered in women with the equal number or more than 102 organisms in HP and in men with an equal number or more than 103 organisms. The collected cultures were assessed from the microbiology sector after determining the strains of each organism, drug resistance by the two methods of disk diffusion and MIC. Antimicrobial sensitivity was assessed for 13 antibiotics such as ciprofloxacin, nitrofurantoin, ampicilin, loxacin, cotrimoxazole, cephalexin, gentamicin, ceftriaxone, nalidixic acid, imipenem, ceftizoxime, co-amoxiclav, and vancomycin.

To identify different strains, the organism grew in differential cultures and the type of strain was determined by adding antiserum and by using the special tables.

The macro tube method was used for MIC. The first solution (stock) was made for its preparation; it was needed to specify the weight of the related antibiotic and diluent fluid volume, which were calculated according to the following formula:

Volume (ml)×concentration (microgr/ ml)Assay potency (microgr/ mg)= (mg) weight of powder Ab

Weight (mg)×Assay potency= (ml) diluent fluid volume

**Concentration**

The antibiotic powder contains a generic nametag and lot number (batch number), the assay potency (the active ingredient of the drug) and the expiring date. The liquid volume is usually calculated at 100ml.

To provide the bacterial suspension, isolated colonies were inoculated in a medium culture of 24-18 hours directly in tubes containing Mueller Hinton, obtaining 0.5 similar turbidities of Mc Farland solution. The prepared Mc Farland: 5.0 Cc (Bacl2 & H20) solution was added to 99.5 Cc sulfuric acid 0.36 normal 0.18 (VN 1%) and then the solution OD was investigated in the wavelength of 625 nm (should be between 0.1–0.08).

From the serial dilutions of the considered antibiotics, 1 cc of each dilution was poured into the sterile screw-cap tube, and 1 cc of bacterial suspension was added. By doubling the final concentration velum in each cell, half of the initially calculated level of considered antibiotics existed. The tubes were incubated for 20-16 hours at 35°C moist heat and the result was read.

The read numbers MIC reported were according to the guidelines of the Clinical and Laboratory Standards Institute [CLSI]. The investigation of drug resistance was calculated by the method of antibiotic disk insertion. A questionnaire was prepared and it recorded the demographic information of patients, the MIC results, and the Antibiogram with the disc. Information using tables and graphs and central indicators and dispersion and chi-square test were analyzed.

## Results

Among the positive urine cultures, 223 cases in which the urinary tract infection was clinically confirmed were tested. There were 131 women (58.7%) and 92 men (41.3 percent). The patients’ age was between 19 and 102 years, with the average age of 0.18 ± 64.8.

The consistency of disk diffusion and MIC tests in the diagnosis of antibiotic sensitivity of isolated organisms was the following:

1. Ciprofloxacin: Disk diffusion sensitivity testing showed a 38.1% sensitivity of the total samples while the sensitivity with the MIC test was 18.8%. With the disc method, it was 60.1% and with the MIC method, 78.5% of the samples were resistant. This difference was statistically significant and indicated that in determining the resistance to ciprofloxacin, the disk diffusion method was not accurate to the MIC method (0.0001 > p and Kappa = 0.292).

The sensitivity and specificity of the disk diffusion test for antibiotic ciprofloxacin, compared to MIC, was calculated according to the related formula and 69% sensitivity and 69.1% specificity were obtained.

2. Co-trimoxazole: disk diffusion testing showed that 24.7% of the samples were sensitive, while the antibiotic sensitivity with the MIC was 7.2%. With the disc method, 75.3% of the samples and with the MIC method, 92.4% showed resistance, which highlighted a better accuracy with the MIC method than with the disk diffusion in the detection of resistance to co-trimoxazole. This difference was statistically significant (p = 0.010).

Sensitivity and specificity of the disk diffusion testing for cotrimoxazole was calculated as it follows 50% sensitivity and 77.3% specificity.

3. Nitrofurantoin: With the disk method, 69.5% of the cases and with the MIC method, 64.6% of the cases were sensitive. The resistance with the disk method was 26.9% and with the MIC method 9.26%. About Nitrofurantoin, the disk diffusion method and the MIC did not present many differences as far as the diagnostic accuracy was concerned. This was statistically significant (0.0001 > p and Kappa = 0.44).

The sensitivity and specificity of the disk diffusion test for nitrofurantoin was calculated as it follows 84.7% sensitivity and 58.2% specificity.

4. Ampicillin: with the disk method, 18.4% of the samples and with the MIC method, 5.4% of cases were sensitive to the drug. With the disc method, 80.7% and with the MIC method, 92.4% of the samples showed resistance. The difference represents a higher accuracy of the MIC method in determining the resistance to ampicilin, being statistically significant (0.0001 > p and Kappa = 0.33).

Sensitivity and specificity of the disk diffusion for ampicilin was calculated as it follows 83.3% sensitivity and 85.3% specificity.

5. Ofloxacin: with the disk method, 28.2% of the samples and with the MIC method, 24.7% of the cases were sensitive to the drug. With the disc method, 69.4% and with the MIC method, 70.4% of the samples showed resistance. That represented the same accuracy of two methods in diagnosing the resistance to drug and was statistically significant (0.0001 > p and Kappa = 0.429).

The sensitivity and specificity of the disk diffusion for ofloxacin was calculated it as follows: 65.5% sensitivity and 83.9% specificity.

6. Cephalexin: with the disk method, 26% of the samples and with the MIC method, 5.8% of the cases were sensitive to the drug. With the disc method 73.1% and with the MIC method, 92.4% of the samples showed resistance. The difference represented a higher accuracy of the MIC method in determining the resistance to the drug and was statistically significant (p = 0.012 and Kappa = 0.116).

The sensitivity and specificity of the disk diffusion for cephalexin was calculated as it follows 46.2% sensitivity and 75.2% specificity.

7. Gentamicin: with the disk method, 43% of the samples and with the MIC method, 30% of the cases were sensitive to the drug. With the disc method 52.5% and with the MIC method, 49.8% of the samples showed resistance. The difference represented a higher accuracy of the MIC method in determining the resistance to drug and was statistically significant (0.0001 > p and Kappa = 0.276).

The sensitivity and specificity of the disk diffusion for gentamicin was calculated as it follows 64.2% sensitivity and 66% specificity.

8. Ceftriaxone: with the disk method, 20.2% of the samples and with the MIC method, 13% of the cases were sensitive to the drug. With the disc method, 78% and with the MIC method, 85.7% of the samples showed resistance. The difference represented a higher accuracy of the MIC method in determining the resistance to the drug and was statistically significant (p = 0.216 and Kappa = 0.074).

The sensitivity and specificity of the disk diffusion for ceftriaxone was calculated as it follows 27.6% sensitivity and 80.9% specificity.

9. Nalidixic acid: with the disk method, 16.1% of the samples and with the MIC method, 17% of the cases were sensitive to the drug. With the disc method 83% and with the MIC method, 82.1% of the samples showed resistance. The difference represented a higher accuracy of the MIC method in determining the resistance to the drug and was statistically significant (0.0001 > p and Kappa = 0.354).

The sensitivity and specificity with the disk diffusion for the nalidixic acid was calculated as it follows 42.1% sensitivity and 89.2% specificity.

10. Imipenem: with the disk method, 40.4% of the samples and with the MIC method, 31.8% of the cases were sensitive to the drug. With the disc method, 57.8% and with the MIC method, 56.5% of the samples showed resistance. The difference represented a higher accuracy of the MIC method in determining the resistance to the drug and the same accuracy in determining the resistance to imipenem and was statistically significant (0.0001 > p and Kappa = 0.306).

The sensitivity and specificity of the disk diffusion for imipenem was calculated as it follows 63.4% sensitivity and 70.4% specificity.

11. Co-amoxiclav: with the disk method, 40.4% of the samples and with the MIC method, 21.1% of the cases were sensitive to the drug. With the disc method, 52.5% and with the MIC method, 70% of the samples showed resistance. The difference represented a higher accuracy of the MIC method in determining the resistance to the drug and was statistically significant (0.0001 > p and Kappa = 0.412).

The sensitivity and specificity of the disk diffusion force-amoxiclav was calculated as it follows 83% sensitivity and 71% specificity.

12. Cefixime: with the disk method, 19.3% of the samples and with the MIC method, 8.5% of the cases were sensitive to the drug. With the disc method, 79.4% and with the MIC method, 89.2% of the samples showed resistance. The difference represented a higher accuracy of the MIC method in determining the resistance to the drug and was statistically significant (0.0001 > p and Kappa = 0.412).

The sensitivity and specificity of the disk diffusion for cefixime was calculated as it follows 21% sensitivity and 80.9% specificity.

13. Vancomycin: with the disk method, 13% of the samples and with the MIC method, 15.2% of the cases were sensitive to the drug. With the disc method 83.9% and with the MIC method, 82.5% of the samples showed resistance. The difference represented the same accuracy of disk and of the MIC method in determining the resistance to Vancomycin and was statistically significant (0.0001 > p and Kappa = 0.532).

The sensitivity and specificity of the disk diffusion for Vancomycin was calculated as it follows 55.9% sensitivity and 94.7% specificity. 

## Discussion

There are a few articles about the sensitivity and specificity of the disk diffusion test and the comparison with the method of determining the minimum inhibitory concentration (MIC) of the drug:

In a survey conducted by Louis et al., the PCR test was conducted on 200 coagulase-negative staphylococci to determine the mecAgene as the standard gold. The sensitivity and specificity of the other tests were calculated as it follows MRSA-Screen 100% and 100%, OXA6 100% and 99%, BMDIL 100% and 60%, E test 100% and 51%, Vitek GPS -SV susceptibility card 98% and 87%, Vitek GPS-107 susceptibility card 100% and 61% [**6**]. However, due to the PCR test, which is a very costly experiment and is not available, this study could only consider the MIC method as a standard gold together with the measurement of sensitivity and specificity of the disk diffusion method.

Marshall conducted a study about the comparison of the sensitivity test of antibiotics (E test, Vitek system, Disk diffusion test). Regarding the standard test of broth micro dilution, on 123 cases of staphylococcus, the following results were obtained: 20.3% strains that were reported with the sensitive conventional method, 15.4% with the E-test method, and 17.9% with the Vitek system method and 17.1% with the Disk diffusion method, all being reported as sensitive. Of 79.7% of the cases that were reported to be resistant with the standard method, 79.7% were with the E test method, 78.9% with the Vitek system method and 79.7% with the Disk diffusion method, all being reported as resistant [**7**]. This article showed the same accuracy of all the above methods and the value of disk diffusion test in determining the antibiotic resistance, of course in our investigations, this consistency being observed in antibiotics such as nitrofurantoin, nalidixic acid vancomycin.

In the survey of Sadighi regarding the sensitivity of the disk diffusion method, two types of the disk of PadtanTeb Company and English Mast Company were studied. The lowest antibiotic sensitivity in both disk diffusion methods was related to co-trimoxazole (PadtanTeb 23% and English Mast 26%), and the highest sensitivity of the antibiotic was related to nitrofurantoin (Iranian drive 86% and English 97%), the study being consistent with our survey [**8**].

In another study of Sadighi about the comparison of the two methods, the disk diffusion and the E-test, in the first method of 100 urinary isolates, sensitive to trimethoprim-sulfamethoxazole, amikacin, ceftriaxone, nalidixic acid, the values were 94%, 66%, 62%, and 52%. In the second method, sensitivity to antibiotics was 37%, 97%, 67%, 50%, respectively. The highest percentage of consistency was between the two methods to amikacin (96%) and the lowest percentage to cotrimoxazole (89%) [**9**].

In a study of Erfanion, 250 samples of E. coli were tested for antimicrobial sensitivity, the testing being conducted by two methods of disk diffusion and E-test with the MIC method, in the case of antibiotics bacteremia, gentamicin, Furadantin, ceftazidime, and ciprofloxacin. About ceftazidime and gentamicin, a 76.8% and 62.2% consistency was reported while for the other antibiotics, 37.8% consistency was observed. This indicated the low sensitivity of the disk diffusion method compared to the minimum inhibitory concentration measurement methods that were consistent with our results [**10**].

## Conclusion

Due to the low responsiveness rates and the specificity of distribution of the disk testing method compared to the MIC method, it seems that the antibiogram should be done with the MIC method in the possibility of drug resistance, particularly in patients with a positive culture regarding the more resistant organisms.

**Acknowledgements**

The authors would like to thank the Deputy of Research and Technology at the University for funding the project and to the honorable microbiology laboratory personnel of Valiasr Hospital for the help offered in finishing the project.
